# The company we keep. Using hemodialysis social network data to classify patients’ kidney transplant attitudes with machine learning algorithms

**DOI:** 10.1186/s12882-022-03049-2

**Published:** 2022-12-29

**Authors:** Rafaa Aljurbua, Avrum Gillespie, Zoran Obradovic

**Affiliations:** 1 Center for Data Analytics and Biomedical Informatics, Temple University, Philadelphia, USA; 2 Department of Computer Science, College of Computer, Qassim University, Buraydah, Saudi Arabia; 3 Division of Nephrology, Department of Medicine, Lewis Katz School of Medicine, Hypertension, and Kidney Transplantation, Temple University, Philadelphia, USA

**Keywords:** Hemodialysis, Kidney transplantation, Social Network, Machine Learning, Psychosocial, Survey Research, Social Determinants of Health

## Abstract

**Background:**

Hemodialysis clinic patient social networks may reinforce positive and negative attitudes towards kidney transplantation. We examined whether a patient’s position within the hemodialysis clinic social network could improve machine learning classification of the patient’s positive or negative attitude towards kidney transplantation when compared to sociodemographic and clinical variables.

**Methods:**

We conducted a cross-sectional social network survey of hemodialysis patients in two geographically and demographically different hemodialysis clinics. We evaluated whether machine learning logistic regression models using sociodemographic or network data best predicted the participant’s transplant attitude. Models were evaluated for accuracy, precision, recall, and F1-score.

**Results:**

The 110 surveyed participants’ mean age was 60 ± 13 years old. Half (55%) identified as male, and 74% identified as Black. At facility 1, 69% of participants had a positive attitude towards transplantation whereas at facility 2, 45% of participants had a positive attitude. The machine learning logistic regression model using network data alone obtained a higher accuracy and F1 score than the sociodemographic and clinical data model (accuracy 65% ± 5% vs. 61% ± 7%, F1 score 76% ± 2% vs. 70% ± 7%). A model with a combination of both sociodemographic and network data had a higher accuracy of 74% ± 3%, and an F1-score of 81% ± 2%.

**Conclusion:**

Social network data improved the machine learning algorithm’s ability to classify attitudes towards kidney transplantation, further emphasizing the importance of hemodialysis clinic social networks on attitudes towards transplant.

**Supplementary Information:**

The online version contains supplementary material available at 10.1186/s12882-022-03049-2.

## Introduction

**Fig. 1:**
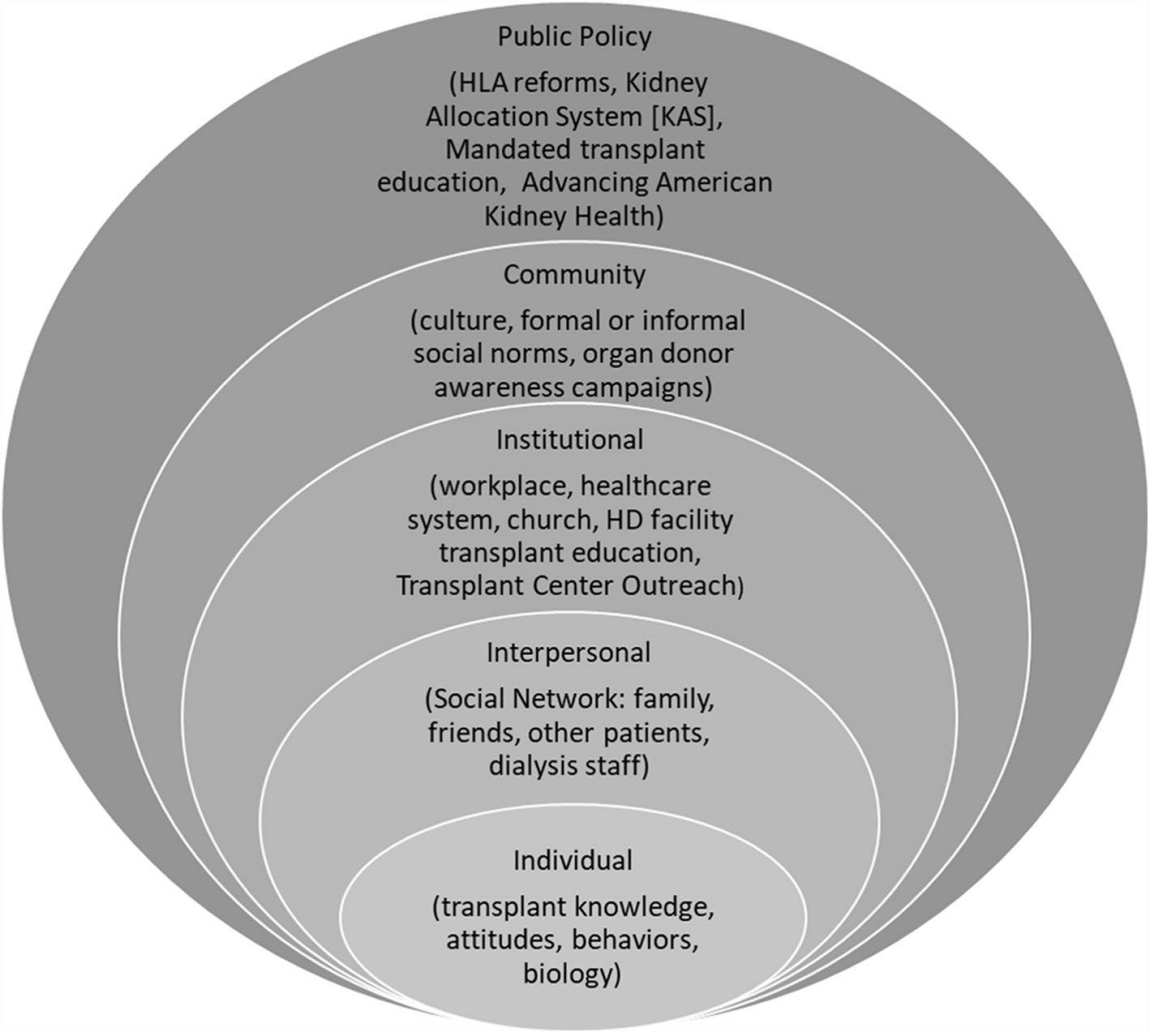
Social-ecological model for kidney transplant disparities. Figure 1. Social-ecological model for kidney transplant disparities is a modified version of two models. [7, 8] The first layer is the individual layer which refers to the patients knowledge, attitudes, behaviors, and biology. This layer is shaped by the other layers of the model. Such as the interpersonal layer (i.e. the individual’s social network), the institutional layer (e.g. the healthcare system), the community layer (e.g. the culture of organ donation and transplant with in the community, the public policy layer (e.g. mandated transplant education). All these layers influence each other and ultimately shape the individual’s beliefs

Kidney transplantation is the optimal treatment choice for end-stage kidney disease (ESKD) yet remains under-utilized in the United States because of barriers to access [1, 2]. These barriers are further exacerbated by extant health disparities and social determinants of health**.** People who are older age, Black race, female sex, of lower education, and of lower income have less access to kidney transplantation [3–6]. Using a social ecological model framework (Fig. 1), [7, 8] most research examining these disparities has focused on 1) how institutions/organizations and public policies affect access in terms of provider bias and structural racism [8] and 2) how community and physical resources affect logistic difficulties of completing medical evaluations. [8, 9] How the interpersonal layer, the relationships that people form (i.e., their social network), influences individual attitudes and behaviors towards kidney transplantation has not been well studied. [10, 11].

Hemodialysis patients’ social networks are unique because in addition to their family and friend networks being a source of potential living donors, hemodialysis patients also form social networks with other patients within the hemodialysis clinic. [12, 13] The hemodialysis clinic social network provides a venue to share information, model behaviors, and reinforce positive and negative attitudes towards kidney transplantation. [12–15] These hemodialysis clinic social networks may contribute to extant disparities if influential network members have negative attitudes towards kidney transplantation and further reinforce other patients’ negative attitudes.

**Fig. 2:**
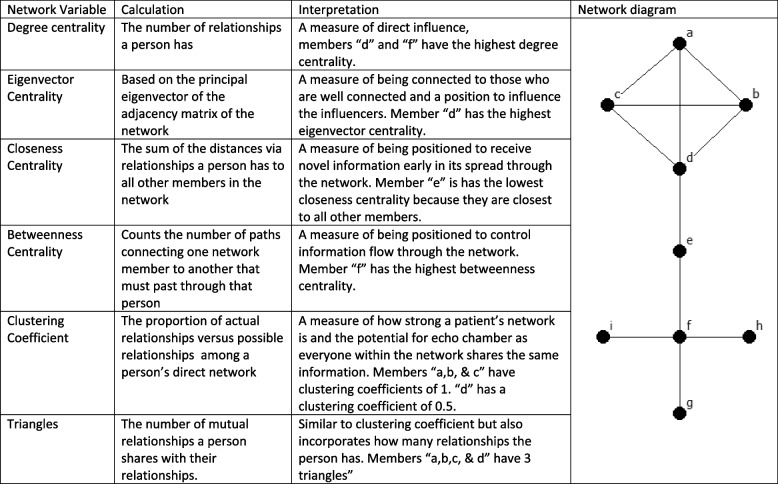
Glossary of Terms. Figure 2. presents a glossary of terms as well as a diagram of a kite network used to demonstrate different centrality measures. Each dot represents a person in the network and they are labelled a-i. A black line represents a relationship between two members of the network

Social network theory posits that a person’s attributes can be predicted by the structure and their position within the network as well as the composition of the social network. [10, 14] Social network analysis is used to measure the structure and composition of the social networks. [14] It is a combination of graph theory, physics, computer science, and sociology, and although uses unique terminology, the concepts tend to be intuitive (Fig. 2). [14, 16] Network structure refers to how interconnected the network members are using the clustering coefficient and the number of triangles formed by the relationships in the network. Network position refers to how central the person is in the network. There are several measures of centrality each with a different interpretation depending on popularity, influence, and access to information (Fig. 2) [16, 17].

We have previously found that patients who formed small densely interconnected networks (clustering coefficient, Fig. 2) within the clinic completed more steps in the transplant process than patients with large networks (degree centrality) or patients who were connected to other patients with large networks (eigenvector centrality). [12] This finding was surprising because people who are central in a network tend to have the greatest access to information [16] and those central in a hemodialysis clinic network should have the most information about transplantation. [10, 14] This was a study of a single clinic’s network, and it remains unknown how network centrality, clustering, and transplant attitudes differ in other hemodialysis clinics’ social networks. Therefore, we decided to study two hemodialysis facilities selected for their geographic and demographic differences with the goal of further demonstrating the association between hemodialysis patients’ social networks and their attitudes toward kidney transplantation while testing the feasibility of machine learning classification algorithms for attitude classification using social network data. [18, 19] By understanding how the hemodialysis clinic social network contributes to patient attitudes, network interventions can be designed promote positive transplant attitudes, improving access, and eliminating disparities in kidney transplantation.

We hypothesize that a patient’s position and local structure within a hemodialysis clinic social network can improve the classification of the patient’s attitudes towards kidney transplantation. In other words, how much can you tell about a person by the company they keep?

## Methods

### Source of data, study design, setting, participants, and survey data collection instrument

This study is a cross-sectional analysis of a baseline social network and transplant attitude survey for the Social Networks and Renal Education [SNARE]: Promoting Transplantation trial, NCT03536858 (25/05/2018). These data were collected between October 2018 and February 2020 in two hemodialysis facilities (in southeastern Pennsylvania and central New Jersey). Data collection for this analysis was not affected by the COVID19 pandemic. These facilities were selected because they were both part of the same dialysis organization but demographically (race and income, Table S1) and geographically (different organ procurement organization, different transplant centers) different.

Patients were eligible to participate if they had end-stage kidney disease (ESKD), spoke English, and were 18 years old or older. The survey was designed to be a census of both dialysis facilities and the anticipated recruitment was 200 participants. Patients were approached and asked to participate in the study during their hemodialysis session. They were asked to participate in a survey about who they talk to about their health and kidney disease both inside and outside of the hemodialysis facility as well as their attitudes towards hemodialysis and kidney transplantation. All data would be kept strictly confidential and not shared with other patients or staff and all results will be deidentified and reported as an aggregate. Patients were excluded if they declined to participate, were unable to give consent or were asleep during the recruitment period, or if they were hospitalized, switched to peritoneal dialysis, received a transplant, transferred out, or died before they could be surveyed. The Temple University Institutional Review Board approved the study protocol; written informed consent was obtained from all participants. The clinical and research activities being reported here are consistent with the Principles of the Declaration of Istanbul as outlined in the “Declaration of Istanbul on Organ Trafficking and Transplant Tourism” as well as adherence to the Declaration of Helsinki [20, 21]. All identifiable data is stored on HIPAA password-protected compliant computers on a secure server in an office that is locked with a key.

We used an interviewer-administered computer-based survey questionnaire for data collection. The questionnaire, which combined three previously validated survey instruments [5, 22, 23]. This questionnaire has two components 1) social network assessment [5, 22, 23] and 2) participants transplant attitudes and sociodemographic and clinical measures. [5, 12, 23].

The social network portion of the questionnaire was designed to identify and quantify the relationships within a hemodialysis patient’s social network. It used three questions to identify patients’ social network members: 1) Who are the patients you talk to? 2) Who are the patients you discuss the effects of kidney disease with? 3) Who are the patients you discuss kidney transplant with? To avoid recall bias, participants were allowed to identify up to twelve other patients which approaches the limit of accurate recall while minimizing cognitive burden [24]. Participants were then asked about the strength of the relationship with each patient they identified using a 10-point scale of emotional intimacy, with 10 being very close and 1 being not close. The interviewer could not tell the participant whether they had been identified by other participants. To protect confidentiality, each patient participant was given a unique numerical identifier by a research coordinator resulting in a social network dataset without identifiable names. Names of patients who did not consent to participate were excluded from this dataset. This deidentified dataset was used for the analysis.

### Outcome variables

The primary outcome was whether the participant had a positive attitude towards transplantation. This was collected by the portion of the questionnaire that assessed participants’ attitudes and communication skills regarding their health and kidney disease [5, 12, 23]. A participant’s kidney transplantation attitude was measured by a survey question that asked, “People have different opinions about kidney transplants. In your opinion, how important is it for you to get a kidney transplant?” [12] A positive attitude was defined by responding to the survey question as extremely or very important. Answering moderately important, somewhat important, or not at all important was considered as having a negative attitude. This dichotomy resulted in a balanced predictor outcome [25].

### Predictor variables

#### Sociodemographic and clinical predictor variables

The independent sociodemographic and clinical variables were treated as categorical (see Table 1). They were collected by the portion of the questionnaire which asked about self-reported health, time on dialysis, and demographic variables such as age, sex, race, income, education level, and marital status [5, 12, 23]. These included age, sex, Black race, marital status, education, employment status, self-reported health, dialysis vintage, dialysis clinic, whether they would accept a living donation, and whether they would accept a deceased donation. These variables were selected as they have been previously shown to be associated with the likelihood of receiving a kidney transplant [3–7].

**Table 1:** Sociodemographic and clinical variables associated with positive and negative attitudes towards transplantation

Demographics N(%)	Positive attitude *N* = 66 (60%)	Negative attitude *N* = 44 (40%)	Total *N* = 110 (100%)	*p* value
Sex				0.64
Female	30 (45.5%)	18 (40.9%)	48 (43.6%)	
Male	36 (54.5%)	26 (59.1%)	62 (56.4%)	
Age				0.04
< 50	16 (24.2%)	6 (13.6%)	22 (20.0%)	
50–59	17 (25.8%)	9 (20.5%)	26 (23.6%)	
60–69	25 (37.9%)	14 (31.8%)	39 (35.5%)	
> 69	8 (12.1%)	15 (34.1%)	23 (20.9%)	
Race				0.05
Black	53 (80.3%)	28 (63.6%)	81 (73.6%)	
Other	13 (19.7%)	16 (36.4%)	29 (26.4%)	
Facility				0.01
1 (Urban)	48 (72.7%)	22 (50.0%)	70 (63.4%)	
2 (Suburban)	18 (27.3%)	22 (50.0%)	40 (36.6%)	
Married/ Cohabit	24 (36.4%)	12 (27.3%)	36 (32.3%)	0.36
Religion				0.78
Protestant	38 (57.6%)	23 (52.3%)	61 (55.5%)	
Catholic	11 (16.7%)	7 (15.9%)	18 (16.4%)	
Jewish	1 (1.5%)	2 (4.5%)	3 (2.7%)	
Muslim	4 (6.1%)	1 (2.3%)	5 (4.5%)	
Other	6 (9.1%)	6 (13.6%)	12 (10.9%)	
None	6 (9.1%)	5 (11.4%)	11 (10.0%)	
Health				0.81
Excellent	1 (1.5%)	0 (0%)	1 (0.9%)	
Very good	9 (13.6%)	6 (13.6%)	15 (13.6%)	
Good	23 (34.8%)	12 (27.3%)	35 (31.8%)	
Fair	26 (39.4%)	20 (45.5%)	46 (41.8%)	
Poor	7 (10.6%)	6 (13.6%)	13 (11.8%)	
Education				0.71
Less than high School	6 (9.2%)	7 (15.9%)	13 (11.9%)	
High School graduate	24 (36.9%)	15 (34.1%)	39 (35.8%)	
College with no degree	10 (15.4%)	6 (13.6%)	16 (14.7%)	
Associate degree	11 (16.9%)	6 (13.6%)	17 (15.6%)	
Bachelor’s degree	12 (18.5%)	6 (13.6%)	18 (16.5%)	
Master's degree	1 (1.5%)	3 (6.8%)	4 (3.7%)	
Ph.D. degree	1 (1.5%)	1 (2.3%)	2 (1.8%)	
Employment				0.13
Employed Full-time	3 (4.5%)	1 (2.3%)	4 (3.6%)	
Employed Part-time	2 (3.0%)	2 (4.5%)	4 (3.6%)	
Unemployed looking for work	3 (4.5%)	0 (0%)	3 (2.7%)	
Unemployed and not looking for work	4 (6.1%)	0 (0%)	4 (3.6%)	
Retired	22 (33.3%)	25 (56.8%)	47 (42.7%)	
Homemaker	1 (1.5%)	0 (0%)	1 (0.9%)	
Disabled	31 (47.0%)	16 (36.4%)	47 (42.7%)	
Income k = $1000				0.05
0-19 k	25 (37.9%)	9 (20.5%)	34 (30.9%)	
20-39 k	12 (18.2%)	8 (18.2%)	20 (18.2%)	
40-59 k	4 (6.1%)	4 (9.1%)	8 (7.3%)	
60-79 k	5 (7.6%)	3 (6.8%)	8 (7.3%)	
80-99 k	4 (6.1%)	0 (0.0%)	4 (3.6%)	
100 k or more	5 (7.6%)	3 (6.5%)	8 (7.3%)	
Don’t know	2 (3.0%)	9 (20.5%)	11 (10.0%)	
Nonresponse	9 (13.6%)	8 (18.2%)	17 (15.5%)	
Dialysis vintage				0.22
< 6 months	3 (4.5%)	0 (0.0%)	3 (2.7%)	
6 months—1 year	9 (13.6%)	3 (6.8%)	12 (10.9)	
1-5 years	29 (43.9%)	26 (59.1%)	55 (50.0%)	
> 5 Years	25 (37.9%)	15 (34.1%)	40 (36.4%)	
Accept kidney from Someone who has died				< 0.001
Yes	64 (97.0%)	25 (58.1%)	89 (81.7%)	
No	2 (3.0%)	17 (39.5%)	19 (17.4%)	
Don’t know	0 (0%)	1 (2.3%)	1 (0.9%)	
Accept kidney from Living donor				< 0.001
Yes	64 (97.0%)	29 (67.4%)	93 (85.3%)	
No	2 (3.0%)	13 (30.2%)	15 (13.8%)	
Don’t know	0 (0%)	1 (2.3%)	1 (0.9%)	
Would You Like More Transplant Information				0.01
Yes	36 (54.5%)	13 (29.5%)	49(44.5%)	
No	30 (45.5%)	31 (70.5%)	61(55.5%)	

Table 1 shows the demographic and clinical data differences between participants who had a positive attitude towards kidney transplantation and those who had a negative attitude. One participant chose not to provide highest level of education

#### Network predictor variables

The network structural measures calculated based upon the results of the social network portion of the survey questionnaire were used as predictors are established measures of network analysis (see Fig. 2) and included degree centrality, eigenvector centrality, closeness centrality, betweenness centrality, and clustering. [14, 16, 17] Degree centrality is the number of relationships a person has and a measure of direct influence. Eigenvector centrality is based on the principal eigenvector of the adjacency social network matrix and a measure of a position to influence the influencers. Closeness centrality is the sum of the distances as measured the number of relationships between a person and all other members in the network. High closeness centrality is a position to receive novel information. Betweenness centrality counts the number of paths connecting one network member to another that must past through that person. People with high betweenness centrality are in a position to control information flow in the network. Clustering coefficient is the proportion of actual relationships in a person’s direct network divided by the total possible relationships. Triangles is the number of mutual relationships a person shares with their network members forming the image of a triangle on the sociogram (see Fig. 2). This measure is similar to clustering coefficient but also incorporates the number of relationships.

#### Missing data

Participants’ surveys were excluded from these analyses if sections of the questionnaire were unanswered or if the survey was less than 90% complete. Non-responses would be coded as such in the dataset or if a patient chose not to answer, it would be coded as 0 (see Table 1).

### Statistical analysis and methods

#### Network statistics

Survey participants who spoke with other survey participants were defined as part of the hemodialysis clinic patient social network. We calculated the degree centrality, eigenvector centrality, closeness centrality, betweenness centrality, number of triangles, and clustering coefficient using an undirected network graph (sociomatrix) weighted for relationship strength.(See Supplemental Methods SM1) The centrality measures were normalized to the mean of each facility.

#### Descriptive statistics

Chi square and Fisher’s exact tests were used to test the statistical significance of independent variables’ associations with categorical dependent variables. For the network variables, *t*-tests with randomization tests were used. [26, 27] (See supplemental Methods SM2).

#### Development of the machine learning classification algorithms

Our primary analysis compared the predictive ability of the logistic regression models to predict transplant attitude based on sociodemographic data, network data and sociodemographic and network data combined. Predicted labels were formed based on a sigmoid function with a 0.5 threshold. Thus, if the probability of a class is greater than the threshold rate, it will be classified as positive, otherwise, it will be classified as negative. Model performance was evaluated in terms of accuracy (Eq. 1), recall (Eq. 2), precision (Eq. 3), and F1-score Eq. (4).

Accuracy= $$\frac{TP + TN}{TP+ FN + TN+ FP} <span class='reftype'>(1)</span>$$ Precision = $$\frac{TP}{TP+FP} <span class='reftype'>(3)</span>$$

Recall = $$\frac{TP}{TP+FN} <span class='reftype'>(2)</span>$$ F1-score = $$2 \times \frac{Precision \times Recall}{Precision + Recall} <span class='reftype'>(4)</span>$$

where TP = true positives, FP = false positives, TN = true negatives, and FN = false negatives. (see Supplemental Methods SM3).

We use fivefold cross validation with four subsections (80%) for training the model and the remaining Sect. (20%) used for validation. Additionally, we split the dataset into two groups, a full dataset with a full number of patients, and a dataset that contained only participants who were part of the hemodialysis social network excluding those who did not talk to other participants (isolates). Moreover, we repeated each experiment five times and report the mean and standard deviations of the experimental results.(see Supplemental Methods SM4).

REDcap (Research electronic data capture) was used for questionnaire administration and data management [28, 29]. SPSS version 25 was used for data processing and descriptive analyses [30]; UCINET was used for t tests with randomization for network variables [27]. Python programming [31] was done in a Jupyter Notebook (software version 6.1.4) [32], and the graph visualization created by Gephi, (software version 0.9.2) [33]. The following Python packages were used: Networkx [34], scikit-learn [35], stellargraph [36], gensim.models [37].

#### Sensitivity analyses

For the first sensitivity analysis, we compared the performance of the model when adding back the patients who were isolates in the network to the models that were based on the network participants only. For the second sensitivity analysis, we tested the performance of the model by separating the dataset by the participant’s facility. For the third analysis, we examined whether support vector machine or neural network models performed better than the logistic regression models.

## Results

### Participant self-reported sociodemographic and clinical data

Table 1 shows the self-reported sociodemographic and clinical data of the 110 patient participants at the two hemodialysis facilities (Figure S1). The response rates were similar at both clinics (57% at facility 1 vs. 53% at facility 2); however, 70 participants were from the urban facility and 42 were from the suburban facility. Over half (56%) of the participants were men. Most participants (74%) identified as Black or African American. The mean age was 60 $\pm$ 13 years old, with 20% being under the age of 50. Age is represented in quartiles for model performance and generalizability. Eighty one percent of participants would accept a deceased donor kidney transplant and 85% would accept a living donor kidney transplant. There were no significant age or sex differences in non-participation (Table S2).

### Description of hemodialysis clinic social networks

**Fig. 3:**
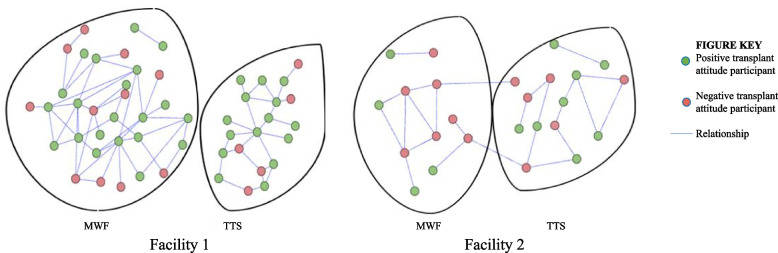
Network graphs of the hemodialysis clinics. Figure 3. Network graphs of the hemodialysis clinics. The green circles (nodes) represent participants with a positive attitude towards transplant and the red nodes represent patients with a negative attitude towards transplant. A blue line (edge) between the participants represents a relationship. The Monday, Wednesday, Friday (MWF) and Tuesday, Thursday, Saturday (TTS) shifts are circled. Note at facility 1 there were no relationships that spanned the MWF and TTS whereas there were two relationships that spanned the MWF and TTS at facility 2

Figure 3 is a visualization of the participants’ hemodialysis facility social networks. MWF represents participants who received treatments on Monday, Wednesday, and Friday and TTS represents participants who received treatments on Tuesday, Thursday, and Saturday (TTS). A green circle represents a participant with a positive transplant attitude, a red circle represents a participant with a negative transplant attitude, and a blue line (link) represents a relationship between participants. Table 2 describes the difference in network statistics between the facilities. Facility 1 (urban southeastern Pennsylvania) had 52 participants in the network with 3 components, 71 links, and 18 participants who were not in the network (isolates). In comparison, facility 2’s (suburban central New Jersey) network had 25 participants in the network with 4 components, 31 links, and 17 isolates. The mean number of relationships among participants (degree) at facility 1 was 2.7, in other words most participants had 2 or more social network members. At facility 2, the mean degree was 2.4. The network members at facility 2 were more interconnected with a mean density was 0.103 or 10.3% of all members were connected and a mean clustering coefficient was 0.32 indicating that 32% of a participant’s network members were connected to each other. In comparison, facility 1 was not as densely interconnected with a density of 0.054 and a mean clustering coefficient of 0.19. Seventy four percent of survey participants at facility 1 were part of the clinic social network and 26% of participants were isolates. At facility 2, 63% of survey participants were part of the social network and 37% were isolates. Isolates are not shown in Fig. 3.

**Table 2:** Network statistics of each clinic

	Facility one	Facility two
Nodes	52	25
Edges	71	31
Average Degree	2.73	2.4
Network Diameter	7	9
Graph Density	0.054	0.103
Connected Components	3	4
Avg. Clustering Coefficient	0.192	0.323
Avg. Path Length	3.105	3.509

Table 2 shows the network statistics of each clinic. Nodes are the number of connected participants in each network. Edges are the number of relationships between the participants. Average degree is the average number of relationships each participant and is weighted for the strength of the relationship with 1 being the strongest relationship to 0.1 the weakest relationship. Network diameter is the maximum distance (number of relationships) between two participants in the network. Graph density is the total number of observed relationships divided by the total possible relationships. Connected components is the number of unique connected networks within in the facilities. Average (Avg.) clustering coefficient is the proportion of relationships among each network members local network. Average (Avg.) path length is the average number of relationships that a network member is connected to any of the other network members (also known as “Degrees of Separation”)

### Attitude towards obtaining a kidney transplant

Sixty-six participants reported that obtaining a kidney transplant was very important or extremely important which we defined as having a positive attitude towards kidney transplantation. The 46 participants who reported that obtaining a kidney transplant was moderately, somewhat, or not at all important were defined as having a negative attitude towards transplantation. Shown in Table 1, participants who had a positive attitude towards kidney transplantation were younger, identified their race as Black or African American, and received hemodialysis at facility 1. The network statistic that was associated with a positive attitude about kidney transplantation was betweenness centrality (Table 3). In other words, participants who served as bridges between other members in the network tended to have a positive attitude towards transplantation.

**Table 3:** Network statistics and attitude towards kidney transplantation

Network Variable	Positive Attitude *N* = 66 Mean (SD)	Negative Attitude *N* = 44 Mean (SD)	*p* value
Degree centrality	0.019 (0.019)	0.014 (0.014)	0.13
Eigenvector Centrality	0.052 (0.102)	0.021 (0.051)	0.08
Closeness Centrality	0.048 (0.040)	0.037 (0.033)	0.14
Betweenness Centrality	0.007 (0.012)	0.003 (0.006)	0.02
Clustering Coefficient	0.148 (0.294)	0.155 (0.308)	0.91
Triangles	0.606 (1.179)	0.523 (1.055)	0.73

Table 3 shows the association between network statistics and attitude towards kidney transplantation. The statistics have been normalized to the statistics of clinic’s network thus the mean value is zero. Degree centrality is the number of relationships a network member has. Eigenvector centrality is how many relationships a network member has to other network member with lots of relationships. Closeness centrality is a measurement of a participant’s distance by relationships to other network members. Betweenness centrality is a measure of how many unique paths between network members must pass through. Clustering Coefficient is the proportion of actual relationships versus possible relationships among a person’s direct network. Triangles is the number of mutual relationships a network member shares with their other network members. The *p* value is calculated via randomization test with 10,000 permutations [27]. Standard Deviation (SD)

### Comparing sociodemographic data to network data in machine learning models to classify participants attitudes towards kidney transplantation

**Fig. 4:**
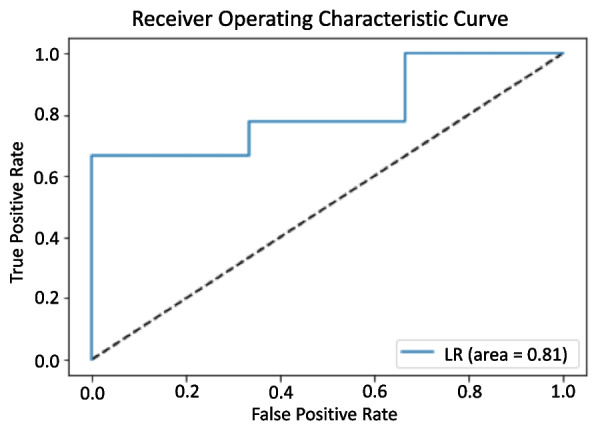
Random Classifier Reciever Operator Curve of Combined Logistic Regression Model. Figure 4. shows the receiver operator of the machine learning combined network, sociodemographic, and clinical data with false positive rate on the x-axis and true positive rate on the the y-axis. LR (logistic regression). [38]

The first analysis included a total of 77 patients who participated in either of the facilities’ social networks (Fig. 3). This analysis (Table 4) compared whether network data, all the variables in Table 3, was better at classifying participants’ attitudes towards kidney transplantation than sociodemographic and clinical data, all the variables in Table 1, using machine learning logistic regression algorithms. The network data model had a higher accuracy, precision, and F1-score than the sociodemographic and clinical data models at classifying attitudes. The network data model obtained an F1-score of 76% ± 2% compared to 70% ± 7% of the sociodemographic and clinical data model. Combining the sociodemographic and the network data had the highest accuracy of 74% ± 3%, a precision of 84% ± 7%, and an F1-score of 81% ± 2% (Table 4). Table 5 shows the top 5 coefficients in the network and the sociodemographic machine learning regression models. Figure 4 shows the area under the curve (AUC) of a random classifier receiver operator curve for the combined sociodemographic and network statistics model. The AUC indicates that there is an 81% chance the model will make a correct prediction.

**Table 4:** Comparing sociodemographic to network variables using machine learning logistic regression

Variables	Accuracy	Precision	Recall	F1-score
Sociodemographic	61% ± 7%	56% ± 9%	95% ± 6%	70% ± 7%
Network statistics data	65% ± 5%	66% ± 6%	90% ± 6%	76% ± 2%
Combined	74% ± 3%	84% ± 7%	79% ± 8%	81% ± 2%

Table 4 shows the results of the machine learning model using sociodemographic/clinical variables and network statistics. Sociodemographic variables included age, sex, Black race, marital status, education, employment status, self-reported health, dialysis vintage, whether they would accept a living donation, and whether they would accept a deceased donation. The network variables included degree centrality, eigenvector centrality, closeness centrality, betweenness centrality, and clustering. The model measure are reported and there standard deviations are reported as percentages

**Table 5:** Top 5 variables in the network and sociodemographic and clinical ML logistic regression models

Variable	Coefficient
**Top 5 Network Variables** the ML Logistic Regression Models	
Eigenvector centrality	0.55
Closeness Centrality	0.51
Degree Centrality	0.47
Betweenness Centrality	0.34
Clustering	0.30
**Top 5 Sociodemographic/Clinical Variables** for the ML Logistic Regression Models	
Would Accept a LDKT	0.87
Would Accept a DDKT	0.82
Health	0.75
Would You like More Transplant Info	0.51
Age	0.49

Table 5 shows the top 5 network variables and sociodemographic/health variables in the machine learning models LDKT; Living donor kidney transplant

### Sensitivity analyses

For the first sensitivity analysis, we compared the performance of the sociodemographic/clinical data and network statistics data using a logistic regression, support vector machine, and neural network models incorporating the participants who were not members of the hemodialysis clinic social networks (isolates, *n* = 33). In general, the network data models including isolates performed better than sociodemographic/clinical data models including isolates.(Figure S2); however, the network data with isolates logistic regression model and sociodemographic and clinical data including isolates logistic regression F1-scores were similar (75% ± 5% vs. 74% ± 7%). The combined logistic regression model, when including isolate participants, still had an F1-score of 80% ± 5%. The logistic regression models outperformed the support vector machine models and neural network models (Figure S2, Table S3). We then examined the performance of the models trained on only one facility (Table S4). For facility 1, the ML logistic regression model F1-score declined to 77% ± 4% and for facility 2 the F1-score declined to 67% ± 4%.

## Discussion

In this study, we mapped the social networks of two geographically and demographically different hemodialysis facilities and found that the hemodialysis facilities’ social networks differed in structure and collective attitudes about kidney transplantation. We utilized these network differences to classify patients’ attitudes towards kidney transplantation. The machine learning models that used network position variables outperformed the models that only used sociodemographic variables associated with negative attitudes towards kidney transplantation [3–6, 8]. This study adds to a growing body of knowledge about the role of hemodialysis patient social networks in shaping the patient’s information, attitudes, and behaviors towards kidney transplantation and further highlighting the promise of hemodialysis social networks and machine learning algorithms to understand and potentially improve access to kidney transplantation.

Following the socioecological model, improving access to transplant should emphasize intervening at the facility level rather than just an individual level. For example, we found that a greater proportion of participants at the urban clinic (facility 1) had a positive transplant attitude than at the suburban clinic (facility 2). These results are similar to those of Browne et al. who found [39], in a census of hemodialysis facilities in the southeastern United States, that different clinics have different collective attitudes towards transplantation. These differences were attributed to the type of transplant information provided by the staff, how the information was delivered, and whether transplant was discussed openly within the clinic by patients and the dialysis facility staff. Differences in clinic norms may explain why previously described sociodemographic variables such as age, race, and socioeconomic status were not strong predictors of transplant attitudes. [3–6, 8] It may not be a matter of cultural or class differences that shape transplant attitudes but rather how information is presented within the dialysis clinics and which norms are established. [15].

Previous network interventions have been developed to disseminate information and change norms and behaviors through social networks. These interventions have mostly focused on reducing smoking and alcohol consumption, exercise and obesity prevention, and public health. [40, 41] These network interventions can be tailored to spread information and modify norms and behaviors within hemodialysis facilities; however, more research is necessary to understand how the hemodialysis facility social networks influence the norms and collective attitudes about transplantation; who is most influential within the social network, and how information is spread within the network. These data presented in this study are a baseline analysis for an ongoing trial examining whether hemodialysis patients central within the hemodialysis clinic network are more likely to disseminate transplant information and behaviors than clustered patients.

Our sample size of two facilities limits the generalizability of this study to other facilities but this study represents a critical step forward because it compares the networks of two facilities in comparison to our previous analysis of a single facility. [12, 15] More hemodialysis clinic social networks need to be mapped. Social network surveys and network analysis tend to be labor intensive, [24] especially given the time needed for this study to recruit and collect data; however, with recent advances in mobile computing and social network software, it is possible to develop a scalable social network mapping tool that can be used by the nephrologist and dialysis staff [42]. Furthermore, as the ML models in this study demonstrate, accurate models can be developed with relatively few survey questions which can streamline future surveys. Future hemodialysis facility interventions could include a shortened form of this survey as part of the annual comprehensive patient assessment. Additionally, despite the rise in social media usage especially since the COVID19 pandemic, [43] little is known about the effects of social media on hemodialysis patients kidney transplant attitudes. [44].

When proposing a novel machine learning algorithm, we must discuss its strength and limitations. In this study, we developed a novel social network-based machine learning algorithm to classify the participant’s attitude towards kidney transplantation. The strength of the dataset used was that it included over half of the patients at both facilities and the surveys were complete without missing data. This sample size was at the limit of a dataset that could be used for machine learning algorithms and the network data model although having higher accuracy and F1 score was not statistically different. Despite our sample size, the combined sociodemographic and network variables model performed quite well with an F1 score of 80% ± 5%. Although the models performed well in two different clinical settings, these models need to be validated in more hemodialysis clinics and will not be clinically applicable until more hemodialysis clinics routinely map the networks of their patients. It is also possible that our definition of a positive transplant attitude was too strict and that patients who thought that obtaining transplant as moderately important should be considered as having a positive attitude. Future research should examine how this attitude changes over time. Lastly, when examining the ethics of this machine learning model, the major focus should be on the social network data. Social network research requires the collection of information on the participant’s social network members who may not have consented to participate in the research. This may raise ethical concerns; however, these data are only a representation of the participant’s perception of their network relationships and this information is not shared with the participant’s network members [45, 46]. Additionally, the model examines the aggregate of the social network excluding non-participants and does not identify other network members specifically.

In conclusion, this study demonstrates the differences in the structures of hemodialysis clinic social networks and the collective attitudes of the members within the networks towards kidney transplantation. Hemodialysis clinic social network data improves the performance of machine learning algorithms to classify patient attitudes about kidney transplantation. In the future, more hemodialysis clinics should have their social networks mapped to identify network interventions to promote and increase access to kidney transplantation.

## Supplementary Information

**Additional file 1:** **Figure S1.** Inclusion and Enrollment in the Study. **Table S1.** Demographic Differences Between Facility 1 and 2. **Table S2.** Age and Sex Differences Between Participants and Non-Participants. (SD) standard deviation. **Figure S2.** Comparing Sociodemographic to Network Variables using Logistic Regression, Support Vector Machine, and Neural Network Models Machine Learning Models including isolates. Sociodemographicvariables included age, sex, Black race, marital status, education, employment status, self-reported health, dialysis vintage, whether they would accept a living donation, and whether they would accept a deceased donation. The network variables included degree centrality, eigenvector centrality, closeness centrality, betweenness centrality, and clustering. The accuracy, precision, recall, and F1-score are the mean of the of running the model five times. They are reported as percentages. The variation of the running the five models are reported in parentheses. **Table S3.** Comparing Sociodemographic to Network Variables using Logistic Regression, Support Vector Machine, and Neural Network Models. **Table S4.** Performance of Machine Learning Algorithm when Data from Only One Facility is Used.

## Data Availability

The datasets generated and analyzed during the current study are not publicly available due to the study is not closed but de-identified (as per the patients’ consent) are available from the corresponding author on reasonable request.

## Abbreviations

Avg: Average
ESKD: End-stage kidney disease
k: $1,000
ML: Machine learning
MWF: Monday, Wednesday, Friday
REDcap: Research electronic data capture
TTS: Tuesday, Thursday, Saturday
