# Supplementary material for: The company we keep. Using hemodialysis social network data to classify patients’ kidney transplant attitudes with machine learning algorithms
Source: BMC Nephrol. 2022 Dec 29;23:414. doi: 10.1186/s12882-022-03049-2 (PMC9798634; doi:10.1186/s12882-022-03049-2)
Supplement: Supplementary file 1 — Additional file 1: Figure S1. Inclusion and Enrollment in the Study. Table S1. Demographic Differences Between Facility 1 and 2. Table S2. Age and Sex Differences Between Participants and Non-Participants. (SD) standard deviation. Figure S2. Comparing Sociodemographic to Network Variables using Logistic Regression, Support Vector Machine, and Neural Network Models Machine Learning Models including isolates. Sociodemographicvariables included age, sex, Black race, marital status, education, employment status, self-reported health, dialysis vintage, whether they would accept a living donation, and whether they would accept a deceased donation. The network variables included degree centrality, eigenvector centrality, closeness centrality, betweenness centrality, and clustering. The accuracy, precision, recall, and F1-score are the mean of the of running the model five times. They are reported as percentages. The variation of the running the five models are reported in parentheses. Table S3. Comparing Sociodemographic to Network Variables using Logistic Regression, Support Vector Machine, and Neural Network Models. Table S4. Performance of Machine Learning Algorithm when Data from Only One Facility is Used. [file 12882_2022_3049_MOESM1_ESM.pdf]

Supplement Table of Contents

Figure S1. Flow chart of inclusion and enrollment in the study…………………………………2

Table S1. Demographic Differences Between Facility 1 and 2……………...…………………...3

Supplemental Methods………………………………………………………………………..4

Table S2. Age and Sex Differences Between Participants and Non-Participants…………………6

Figure S2. Comparing Sociodemographic to Network Variables using Machine Learning Models including Network Isolates…………………………………………………....…………...7

Table S3. Comparing Sociodemographic to Network Variables using Logistic Regression, Support Vector Machine, and Neural Network Models…………………………………………..8

Table S4. Performance of Machine Learning Algorithm when Data from Only One Facility is Used……………………………………………………………………………………………9

Figure S1. Inclusion and Enrollment in the Study

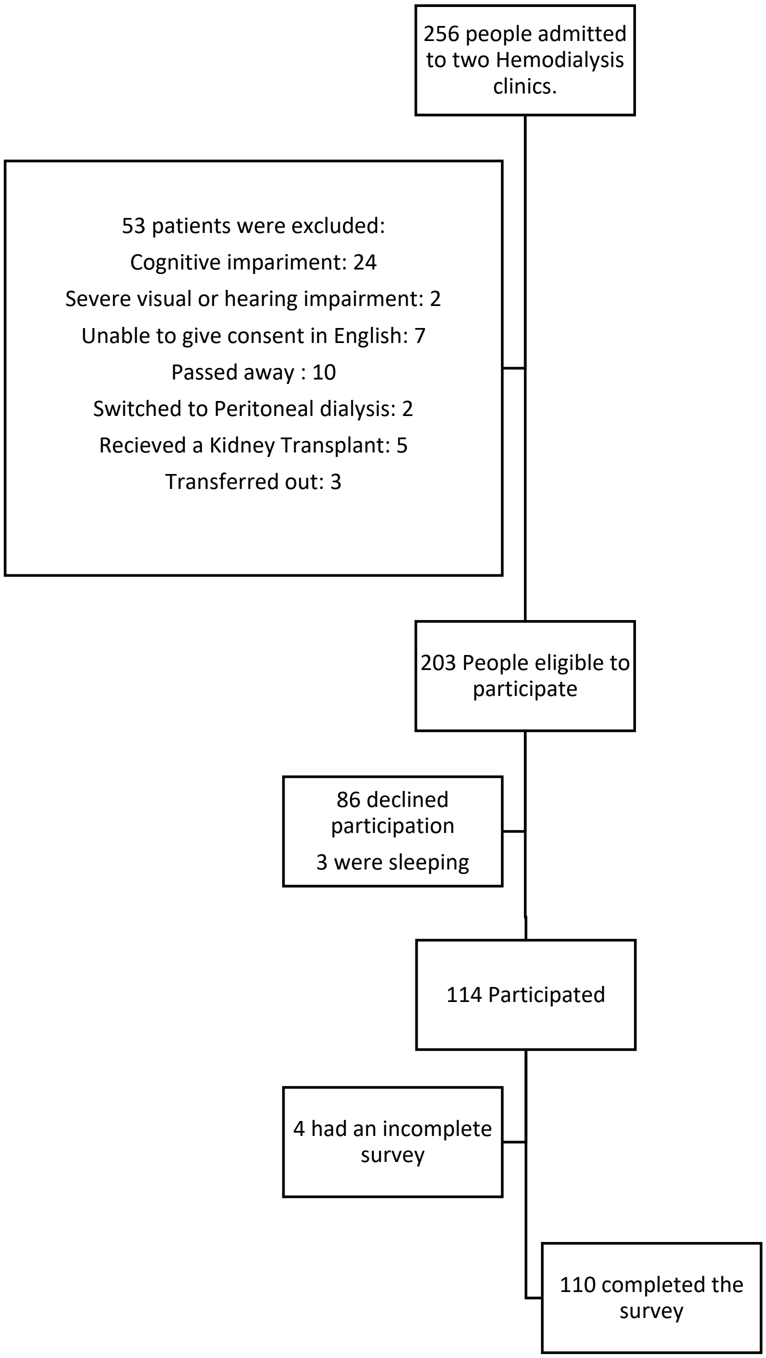

Table S1. Demographic Differences Between Facility 1 and 2.

Demographics N(%)	Facility 1, N = 70 (64%)	Facility 2, N= 40 (36%)	*p* value
Sex			0.17
Female	36 (51.4%)	26 (65.0%)	
Male	34 (48.6%)	14 (35.0%)	
Age			0.15
< 50	15 (21.4%)	7 (17.5%)	
50-59	19 (27.1%)	7 (17.5%)	
60-69	26 (37.1%)	13 (32.5%)	
>69	10 (14.3%)	13 (32.5%)	
Race			<0.001
Black	65 (7.1%)	16 (40.0%)	
Other	5 (92.9%)	24 (60.0%)	
Income k=$1000			<0.001
0-19k	31 (44.3%)	3 (7.5%)	
20-39k	16 (22.9%)	4 (10.0%)	
40-59k	4 (5.7%)	4 (10.0%)	
60-79k	4 (5.7%)	4 (10.0%)	
80-99k	3 (4.3%)	1 (2.5%)	
100k or more	2 (2.9%)	6 (15.0%)	
Don’t know	4 (5.7%)	7 (17.5%)	
Nonresponse	6 (8.6%)	11 (27.5%)	

Table S1 shows the demographic between participants at Facility 1 (urban) and Facility 2 (suburban).

Supplemental Methods

SM1 Network Statistics

All identified relationships were then compiled into an adjacency matrix which was used for calculating network statistics and network visualizations. Relationships were treated as undirected, and the strength of the reciprocated relationships were computed. If two participants identified the same relationship but reported different strengths of the relationship, the mean of the relationship strength was used. Relationship strength was transformed to a weight by dividing the strength by 10, with 1 being very close and 0.1 being not close. This resulted in an undirected network graph weighted by relationship strength for each facility. because centrality measures are highly dependent on the size of the network and would be skewed towards the larger network if the networks are not equal in size.

SM2 Randomization Test

The randomization test is recommended in social network analyses because the social network variables cannot be presumed to be independent (26). To counter the increased risk of type 1 error when using network statistics, the randomization tests used a bootstrapping method, which samples 10,000 random permutations of the observed network variables to estimate standard errors (27).

SM3 Model Accuracy

Accuracy is measured as the percentage of correct predictions for the test data. Recall is the measure of the model correctly identifying true positive cases. Precision is the ratio of the true positive cases to all positive cases. The F1-score is defined as the measure of both the tests precision and recall.

SM4 Machine Learning Models

After the sociodemographic, clinical, and network variables (features) were selected, we applied one-hot encoding that represents the participant's data in the form of binary vectors. We conducted our classification experiments by training and evaluating logistic regression models.

Logistic regression machine learning models were applied to 1) a model based on the sociodemographic/clinical variables and 2) a model based on the participant's network structural variables. We then compared the performance of the models. Next, we combined sociodemographic variables with the network variables and examined the performance of this classification model. These models were first applied to study participants who participated in the social network, excluding isolates. Then the models were used for all participants including isolates as part of the sensitivity analysis.

Table S2. Age and Sex Differences Between Participants and Non-Participants. (*SD*) standard deviation

	Sample N=110	Population of Both Centers N= 256	*p* value
Age mean (*SD*)	60 (13)	62 (14)	0.20
Female Sex	48%	44%	0.48

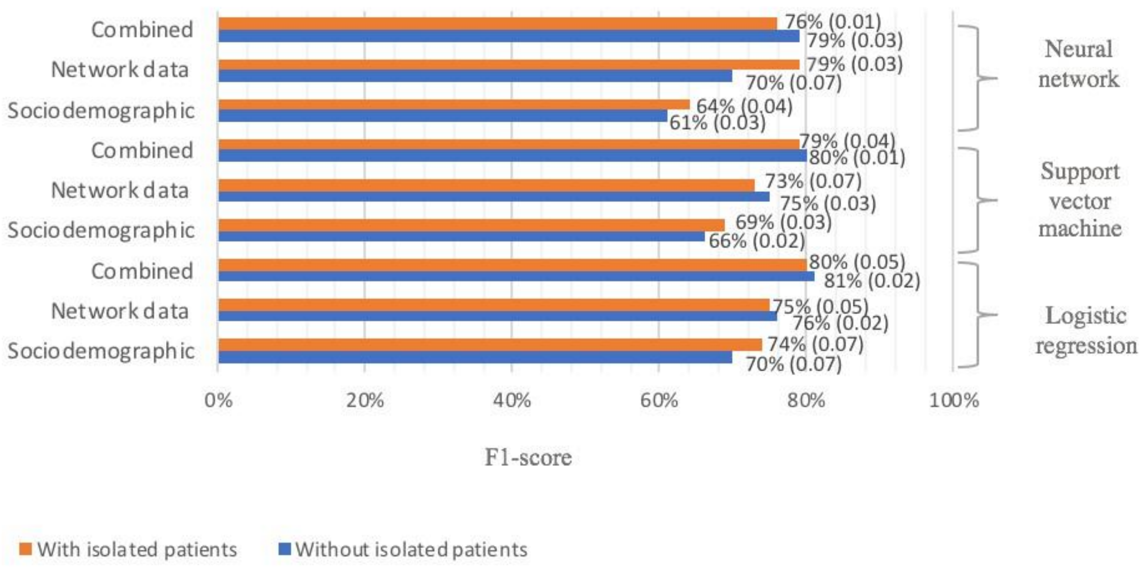

Figure S2. Comparing Sociodemographic to Network Variables using Logistic Regression, Support Vector Machine, and Neural Network Models Machine Learning Models including isolates. Sociodemographic variables included age, sex, Black race, marital status, education, employment status, self-reported health, dialysis vintage, whether they would accept a living donation, and whether they would accept a deceased donation. The network variables included degree centrality, eigenvector centrality, closeness centrality, betweenness centrality, and clustering. The accuracy, precision, recall, and F1-score are the mean of the of running the model five times. They are reported as percentages. The variation of the running the five models are reported in parentheses.

Table S3. Comparing Sociodemographic to Network Variables using Logistic Regression, Support Vector Machine, and Neural Network Models.

Variables	Model	Accuracy	Precision	Recall	F1-score
Sociodemographic	Logistic regression	61% (7%)	56% (9%)	95% (6%)	70% (7%)
	Support vector machine	57% (1%)	63% (5%)	71% (9%)	66% (2%)
	Neural network	52% (7%)	48% (6%)	84% (5%)	61% (3%)
Network statistics data	Logistic regression	65% (5%)	66% (6%)	90% (6%)	76% (2%)
	Support vector machine	63% (6%)	64% (8%)	92% (8%)	75% (3%)
	Neural network	60% (4%)	60% (8%)	86% (7%)	70% (7%)
Combined	Logistic regression	74% (3%)	84% (7%)	79% (0.08)	81% (2%)
	Support vector machine	72% (3%)	76% (7%)	85% (9%)	80% (0.01)
	Neural network	70% (6%)	66% (5%)	96% (5%)	79% (3%)

Table S3 comparing the performance of sociodemographic to network variables using Logistic Regression, Support Vector Machine, and Neural Network Models. Sociodemographic variables included age, sex, Black race, marital status, education, employment status, self-reported health, dialysis vintage, whether they would accept a living donation, and whether they would accept a deceased donation. The network variables included degree centrality, eigenvector centrality, closeness centrality, betweenness centrality, and clustering. The accuracy, precision, recall, and F1-score are the mean of the of running the model five times. They are reported as percentages. The variation of the running the five models are reported in parentheses.

Table S4. Performance of Machine Learning Algorithm when Data from Only One Facility is Used

	Accuracy		Precision		Recall		F1	
	Facility 1	Facility 2	Facility 1	Facility 2	Facility 1	Facility 2	Facility 1	Facility 2
Sociodemographic	61% (3%)	53% (6%)	68% (6%)	54% (6%)	67% (7%)	72% (5%)	66% (1%)	62% (2%)
Network statistics data	56% (5%)	52% (6%)	56% (5%)	48% (2%)	98% (4%)	93% (6%)	70% (5%)	63% (0.02)
Combined	70% (7%)	59% (4%)	77% (2%)	60% (5%)	78% (9%)	77% (3%)	77% (4%)	67% (4%)

Table S4. The results of the machine learning logistic regression models using the sociodemographic variables, the network statistics, the combination of both, the combined model by each facility. The dataset was separated by clinical site and trained on that subset. Sociodemographic variables included age, sex, Black race, marital status, education, employment status, self-reported health, dialysis vintage, whether they would accept a living donation, and whether they would accept a deceased donation. The network variables included degree centrality, eigenvector centrality, closeness centrality, betweenness centrality, and clustering. The accuracy, precision, recall, and F1-score are the mean of the of running the model five times. They are reported as percentages. The standard deviation from running the five models are reported in parentheses as percentages.
